# Development of a Dynamic Nomogram for Predicting the Probability of Satisfactory Recovery after 6 Months for Cervical Traumatic Spinal Cord Injury

**DOI:** 10.1111/os.13679

**Published:** 2023-02-13

**Authors:** Xin Yan, Yaozhi He, Mengxian Jia, Jiali Yang, Kelun Huang, Peng Zhang, Jiaxin Lai, Minghang Chen, Shikang Fan, Sheng Li, Ziwei Fan, Honglin Teng

**Affiliations:** ^1^ Department of Spine Surgery The First Affiliated Hospital of Wenzhou Medical University Wenzhou China; ^2^ Department of Pediatric Allergy and Immunology The Second Affiliated Hospital and Yuying Children's Hospital of Wenzhou Medical University Wenzhou China

**Keywords:** Cervical traumatic spinal cord injury, Nomogram, Prognosis, Risk factors

## Abstract

**Objective:**

Cervical traumatic spinal cord injury (CTSCI) is a seriously disabling disease that severely affects the physical and mental health of patients and imposes a huge economic burden on patients and their families. Accurate identification of the prognosis of CTSCI patients helps clinicians to design individualized treatment plans for patients. For this purpose, a dynamic nomogram was developed to predict the recovery of CTSCI patients after 6 months.

**Methods:**

We retrospectively included 475 patients with CTSCI in our institution between March 2013 and January 2022. The outcome variable of the current study was a satisfactory recovery of patients with CTSCI at 6 months. Univariate analyses and univariate logistic regression analyses were used to assess the factors affecting the prognosis of patients with CTSCI. Subsequently, variables (*P* < 0.05) were included in the multivariate logistic regression analysis to evaluate these factors further. Eventually, a nomogram model was constructed according to these independent risk factors. The concordance index (C‐index) and the calibration curve were utilized to assess the model's predictive ability. The discriminating capacity of the prediction model was measured by the receiver operating characteristic (ROC) area under the curve (AUC). One hundred nine patients were randomly selected from 475 patients to serve as the center's internal validation test cohort.

**Results:**

The multivariate logistic regression model further screened out six independent factors that impact the recovery of patients with CTSCI. Including admission to the American Spinal Injury Association Impairment Scale (AIS) grade, the length of high signal in the spinal cord, maximum spinal cord compression (MSCC), spinal segment fractured, admission time, and hormonal therapy within 8 h after injury. A nomogram prediction model was developed based on the six independent factors above. In the training cohort, the AUC of the nomogram that included these predictors was 0.879, while in the test cohort, it was 0.824. The nomogram C‐index incorporating these predictors was 0.872 in the training cohort and 0.813 in the test cohort, while the calibration curves for both cohorts also indicated good consistency. Furthermore, this nomogram was converted into a Web‐based calculator, which provided individual probabilities of recovery to be generated for individuals with CTSCI after 6 months and displayed in a graphical format.

**Conclusion:**

The nomogram, including ASIA grade, the length of high signal in the spinal cord, MSCC, spinal segment fractured, admission time, and hormonal therapy within 8 h after injury, is a promising model to predict the probability of content recovery in patients with CTSCI. This nomogram assists clinicians in stratifying patients with CTSCI, enhancing evidence‐based decision‐making, and individualizing the most appropriate treatment.

## Graphical abstract

We retrospectively included 475 patients with traumatic cervical spinal cord injury (CTSCI) at our hospital between March 2013 and January 2022. Six independent influencing factors were selected, a nomogram was created and validated for its predictive ability.

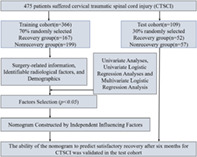

## Introduction

Cervical traumatic spinal cord injury (CTSCI) accounts for around 55% of all traumatic spinal cord injuries (SCI).^1^ CTSCI is a severely disabling condition that frequently results in sensory and motor dysfunction below the level of injury, which significantly impacts the patient's physical and mental health and places a tremendous economic burden on the patient's family and society.^2^, ^3^ Accurate identification of factors affecting the prognosis of patients with CTSCI can help clinicians design individualized treatment plans for patients and treat them with interventions as soon as possible, which is essential to improve their quality of life later in life.

A clinician must be able to accurately anticipate the likelihood of a patient recovering from a CTSCI to give the highest quality of care to patients and their families throughout the disease's progression.^4^ Several studies have reported on the potential factors influencing neurological or functional recovery after CTSCI. Maximum spinal cord compression (MSCC) and the admission American Spinal Injury Association Impairment Scale (AIS) grade were proved as significant predictors of spinal cord function in patients with CTSCI.^5^, ^6^ In addition, studies have highlighted possible influencing factors, including age, damage segment, admission time, and hormonal therapy within 8 h after injury.^7^, ^8^

In recent years, nomograms have been widely used for prognostic studies and risk assessment of cancer.^9^, ^10^ Nomograms are an effective form of study in translational medicine that converts simple data into visual, user‐friendly graphs through mathematical modeling and visual risk assessment, where clinicians can more vividly present their predictions of future events to patients rather than cursory reporting of the corresponding risk factors.^11^ It is gaining increasing popularity with clinicians due to its convenience, precision, vividness, and user‐friendliness and is gradually becoming an alternative to traditional scoring scales and even a new standard.^12^

Unfortunately, no prognostic nomogram for patients with CTSCI has been developed up to date. Based on these considerations, this study reviewed the clinical and imaging data of CTSCI patients at our institution to analyze the impact of these factors on the neurological recovery of CTSCI patients after 6 months. Therefore, the purpose of this study was: (i) identify independent influences on the prognosis of patients with CTSCI; and (ii) establish and evaluate a novel clinical model to predict the probability of neurological recovery in CTSCI patients after 6 months.

## Methods

### Participants

We retrospectively included 475 patients with CTSCI in our institution between March 2013 and January 2022. Three hundred sixty six patients were randomly selected as the training cohort and the remaining 109 patients as the test cohort.

The following were the inclusion criteria: (i) CTSCI; (ii) computed tomography (CT) and magnetic resonance images (MRI) of the cervical spine within 24 h of admission; (iii) complete data including pre‐treatment AIS grade, pre‐treatment Japanese Orthopaedic Association (JOA) score, JOA score 6 months after injury; and (iv) completion of 6 months follow‐up. The exclusion criteria were as follows: (i) combined moderate to severe craniocerebral trauma, thoracic and abdominal organ injuries, severe fractures of extremities; (ii) combined spinal cord injury of the thoracolumbar region; (iii) merge obviously collapsed vertebrae; (iv) recurrence of CTSCI within 6 months; (v) non‐traumatic disease in the spinal cord, such as infection or tumor; (vi) combined dementia, Parkinson's disease, nerve damage to extremities; (vii) Prior systemic disorders include renal failure, cancer, liver cirrhosis, or heart failure; (viii) missing or insufficient information; and (ix) death or loss of visitation within 6 months after injury. The ethical review board of the First Affiliated Hospital of Wenzhou Medical University granted approval to conduct our study (KY2022‐R154).

### Variables

Our institution's medical records gathered variables, including surgery‐related information, identifiable radiological factors, and demographics. Radiological identifiable factors included the length of high signal in the spinal cord, MSCC, increased signal intensity (ISI), damage segment, highest damaged segment, ossification of the posterior longitudinal ligament (OPLL), the number of sagittal T2‐WI MRI image, and spinal segment fractured. Demographic and surgery‐related information includes age, sex, BMI, smoking, drinking, diabetes, hypertension, hyperlipidemia, Vitamin D, trauma energy, AIS grade, pre‐treatment JOA, and JOA score 6 months after injury, admission time, hormonal therapy within 8 h after injury, and rehabilitation.

### Radiological Evaluation

Two certified orthopedic surgeons with a master's degree in spinal research read the films and recorded the data, and the quantitative information was averaged from the data measured. The length of high signal in the spinal cord was determined.

### Maximum Spinal Cord Compression (MSCC)

**Fig. 1:**
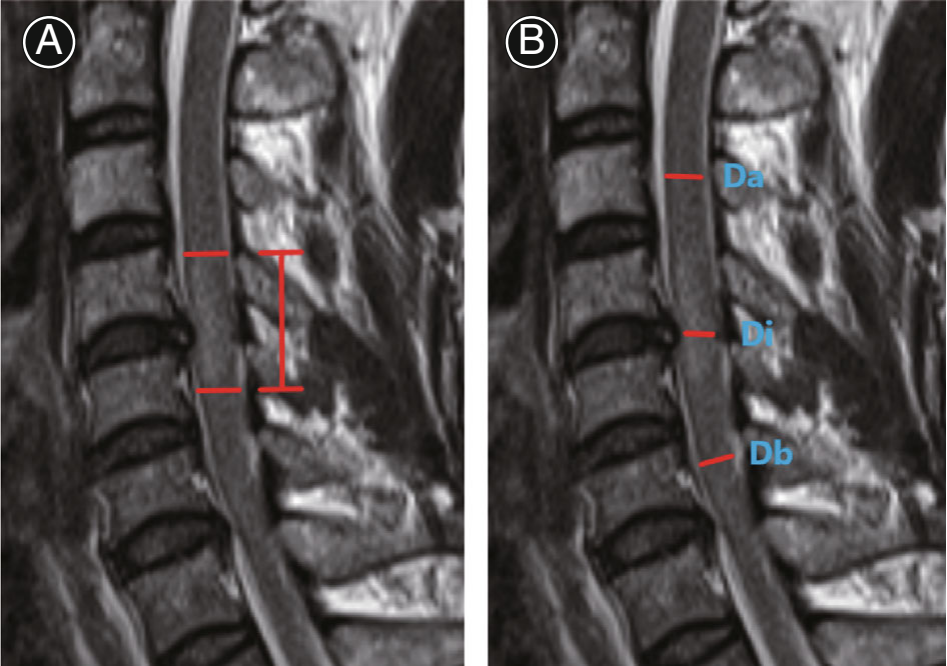
(A) Vertical length of high signal in spinal cord measured as the distance between the most cranial and most distal point of spinal cord edema in the mid‐sagittal plane on T2‐WI. (B) Based on the cord diameter at the level of maximum stenosis (Di), the normal level cord diameter closest to the level of stenosis (Da) and the cord diameter closest to the normal level at the caudal end of stenosis (Db) on T2‐weighted sagittal MR images. The maximum cord compression was also calculated as follows: Maximum spinal cord compression (%) = [1–2 × Di/(Da + Db)] × 100%

The sagittal diameter of the spinal cord at maximum compression (Di) and the sagittal diameter of the spinal cord at one segment above and below the injured segment (Da, Db) was measured as previously described by Miyanji *et al*.^5^ and Fehlings *et al*.^13^ on a median sagittal T2‐weighted and the MSCC were calculated according to their formula, MSCC = [1–2 × Di/(Da + Db)] × 100% (Fig. 1).

### Increased Signal Intensity (ISI)

**Fig. 2:**
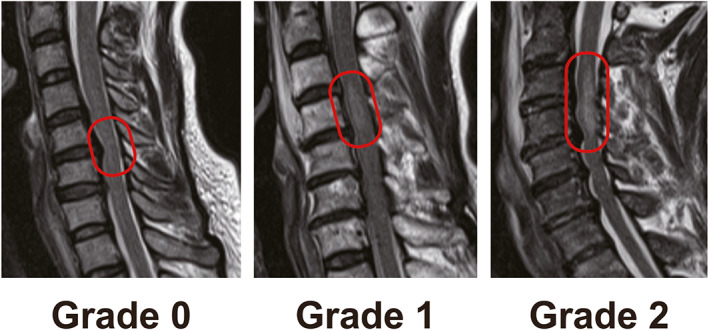
Classification of ISI of the spinal cord in T2‐weighted magnetic resonance images. ISI was classified into three grades: Grade 0, none; Grade 1, light (obscure); and Grade 2, intense (bright). ISI, increased signal intensity

Increased signal intensity (ISI) of the spinal cord on T2‐WI MRI was classified into three grades: grade 0, none; grade 1, light (obscure); and grade 2, intense (bright), according to the method of Yukawa *et al*.^14^ (Fig. 2).

### Damage Segment

**Fig. 3:**
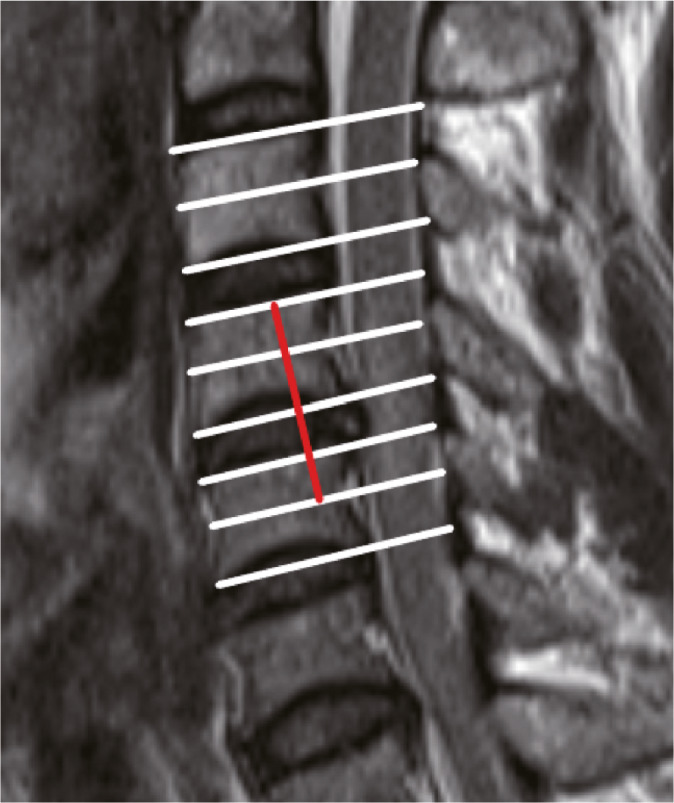
Extent of cord damage was measured by the number of segments

Each vertebral body was divided into two segments, upper and lower, and the lower intervertebral disc was defined as a segment, according to the method proposed by Nakajima *et al*.^15^ and the number of segments in the area of signal enhancement was recorded on T2‐WI MRI (Fig. 3).

### Highest Damaged Segment

**Fig. 4:**
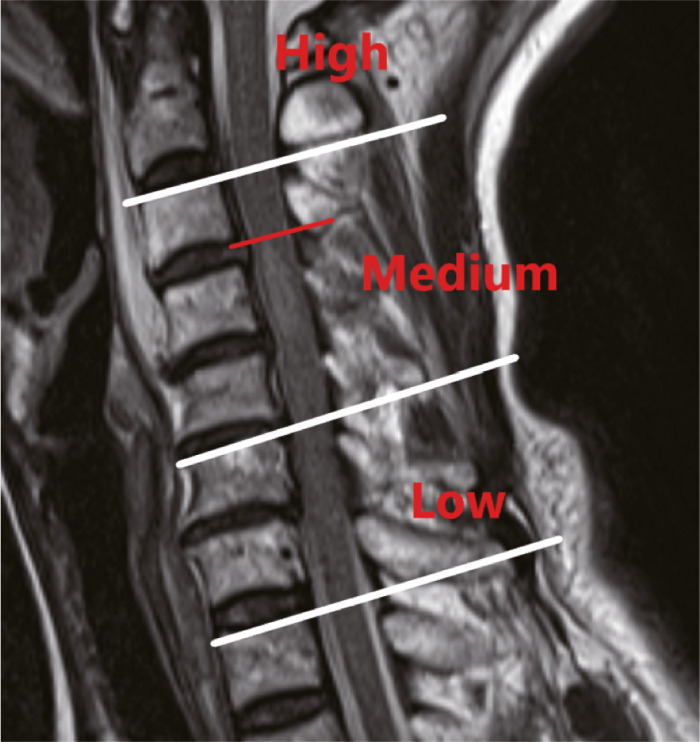
T2‐WI MRI showing edema in the median sagittal plane at the headmost part of the spinal cord above the superior endplate of C3 was defined as a high injury, and edema from the superior endplate of C3 to the superior endplate of C6 was defined as a medium injury, and edema from the superior endplate of C6 to the superior endplate of T1 was defined as a low injury

T2‐WI MRI showing edema in the median sagittal plane at the headmost part of the spinal cord above the superior endplate of C3 was defined as a high injury, and edema from the superior endplate of C3 to the superior endplate of C6 was defined as a medium injury, and edema from the superior endplate of C6 to the superior endplate of T1 was defined as a low injury (Fig. 4).

### OPLL and Spinal Segment Fractured

**Fig. 5:**
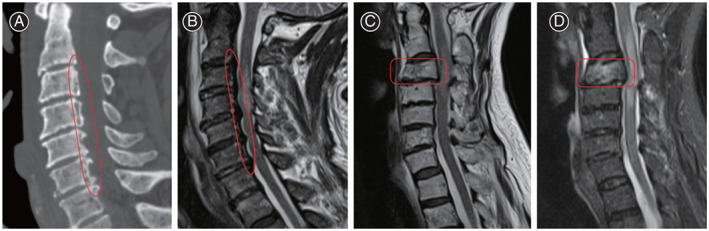
(A, B) CT and MRI sagittal scan demonstrated spinal stenosis from C3 to C6 and severe OPLL from C3 to C6. The red solid oval shows OPLL. (C, D) Sagittal scans on T2 and STIR sequences on MRI showed a fracture in C3 and a strong high intensity suggestive of a fresh fracture in C3 on STIR sequences. The red solid line square shows fresh fractures (C3). OPLL, ossification of the posterior longitudinal ligament

Differentiating patients with OPLL and fractured spinal segment based on CT and MRI (Fig. 5).

### Trauma Energy

Trauma energy is classified according to their energy level as high energy or low energy, high energy including injuries from car accidents and falls from heights, and low energy including falls on foot.

### Clinical Evaluation

All patients admitted to the hospital were assessed for spinal cord function using the American Injury Scale (AIS) and the Japanese Orthopaedic Association (JOA) score. JOA scores were obtained at 6 months by outpatient or telephone follow‐up. Injuries are classified according to their energy level as higher‐energy trauma and low‐energy trauma, higher‐energy trauma including a traffic accident and high fall (>1 m in height), and low‐energy trauma including fall on a level surface and low fall (<1 m in height). The rate of improvement in the JOA score was calculated according to the method of Hirabayashi *et al*.^16^ [(JOA score at last follow‐up − admission JOA score)/(17 − admission JOA score) × 100%]. JOA score improvement rate > 50% was defined as a satisfactory prognosis, and JOA scores improvement rate ≤ 50% was defined as a poor prognosis, according to Yamazaki *et al*.^17^

### Statistical Analysis

Categorical variables were expressed as percentages and tested using the χ2 or Fisher's exact test. Continuous variables were shown as mean ± standard deviation or median with the interquartile range. Continuous variables with normal or non‐normal distributions were examined using the Shapiro–Wink (SW) statistic. A *t*‐test was used for normally distributed continuous variables, and a U‐test was used for non‐normally distributed continuous variables. Univariate logistic regression analysis was then performed. To identify independent risk factors, significant variables with *P*‐values < 0.05 were included in a multifactorial logistic regression analysis. A forest plot was utilized to visualize the results of univariate and multivariate regression studies. Ultimately, a nomogram model was developed based on the independent risk variables eliminated using multivariate logistic regression.

We constructed the ROC and the AUC to measure the specificity and sensitivity of the model and its discriminatory ability, respectively, with values closer to one indicating good predictive discriminatory ability.^3^ Both the calibration curve and the concordance index (C‐index) were utilized in the evaluation of the performance of the prediction model. Integration of the calibration curve was performed to assess the nomogram's precision. The C‐index, which may range anywhere from 0.5 to 1.0, is utilized to determine how discriminating the model is, and a larger C‐index reveals more accurate results regarding differentiating subjects. After that, a corrected C‐index was computed using the bootstrapping validation (1000 bootstrap resamples), which was done to validate the correctness of the model once again. R version 4.1.2 for Windows (R Foundation for Statistical Computing, Vienna, Austria) and GraphPad Prism 8 software (GraphPad Software Inc., San Diego, CA) were used to conduct statistical analysis. The DynNom and Shiny packages were utilized to produce an online calculator (https://www.shinyapps.io/). All statistical tests were two‐tailed, and *P*‐values < 0.05 were regarded as statistical significance.

## Results

### Study Population

In the training cohort, a total of 366 CTSCI patients (280 males; 86 females) were included in this study. The mean age at the time of injury was 59.00 [50.00, 67.00] years, and most victims were male (76.5%). The most common cause of cervical spine injury was traffic accident 148 (40.44%), fall on level surface 119 (32.51%), and high fall (>1 m in height) 99 (27.05%). The AIS scores were 67 (18.3%) for grade A, 46 (12.6%) for grade B, 132 (36.1%) for grade C and 121 (33.1%) for grade D. On admission, 282 (77.0%) patients underwent surgery and 84 (23.0%) patients received continuous conservative treatment. Of these CTSCI patients, 167 patients achieved satisfactory recovery of neurological function. Information on patients in the training cohort is shown in Table 1.

**TABLE 1:** Comparison of demographic, clinical and laboratory characteristics of CTSCI patients according to neurological satisfactory recovery in the training cohort

Variable	Total	Nonrecovery group	Recovery group	Z/χ2	*P* value
Number^b^	366 (100)	199 (54.37)	167 (45.63)		
Age (years)^a^	59.00 (50.00, 67.00)	60.00 (50.50, 68.00)	56.00 (48.50, 66.00)	−1.578	0.115
BMI (kg/m^2^)^a^	24.05 (22.16, 26.17)	24.22 (22.31, 26.62)	23.83 (22.00, 25.39)	−1.627	0.104
The length of high signal (mm)^a^	14.56 (6.42, 29.72)	24.96 (11.00, 43.00)	8.52 (4.92, 14.80)	−8.378	<0.001***
MSCC^a^	28.45 (16.75, 37.74)	33.39 (25.22, 40.67)	20.84 (11.19, 30.11)	−7.274	<0.001***
Vitamin D (μg)^a^	58.30 (47.32, 71.69)	56.04 (45.60, 69.11)	59.67 (49.12, 73.64)	−2.14	0.032*
The number of sagittal T2‐WI MRI image^a^	5.00 (3.00, 5.00)	5.00 (4.00, 5.00)	5.00 (3.00, 5.00)	−1.803	0.071
Sex^b^				0.004	0.949
Female	86 (23.50)	46 (23.12)	40 (23.95)		
Male	280 (76.50)	153 (76.88)	127 (76.05)		
Trauma energy^b^				27.02	<0.001***
Low‐energy trauma	119 (32.51)	41 (20.60)	78 (46.71)		
Fall on level surface	119 (32.51)				
Higher‐energy trauma	247 (67.49)	158 (79.40)	89 (53.29)		
Traffic accident	148 (40.44)				
High fall (>1 m in height)	99 (27.05)				
AIS grade^b^				−9.756	<0.001***
A	67 (18.31)	64 (32.16)	3 (1.80)		
B	46 (12.57)	33 (16.58)	13 (7.78)		
C	132 (36.07)	73 (36.68)	59 (35.33)		
D	121 (33.06)	29 (14.57)	92 (55.09)		
ISI^b^				−4.464	<0.001***
Grade 0	44 (12.02)	12 (6.03)	32 (19.16)		
Grade 1	297 (81.15)	167 (83.92)	130 (77.84)		
Grade 2	25 (6.83)	20 (10.05)	5 (2.99)		
Damage segment^b^				−8.865	<0.001***
<3 segments	216 (59.02)	77 (38.69)	139 (83.23)		
3–9 segments	109 (29.78)	83 (41.71)	26 (15.57)		
>9 segments	41 (11.20)	39 (19.60)	2 (1.20)		
Highest damaged segment^b^				−5.885	<0.001***
High	38 (10.38)	34 (17.09)	4 (2.40)		
Medium	268 (73.22)	148 (74.37)	120 (71.86)		
Low	60 (16.39)	17 (8.54)	43 (25.75)		
OPLL^b^				3.64	0.056
NO	234 (63.93)	118 (59.30)	116 (69.46)		
YES	132 (36.07)	81 (40.70)	51 (30.54)		
Admission time^b^				−3.677	<0.001***
<3 h	148 (40.44)	69 (34.67)	79 (47.31)		
3–8 h	161 (43.99)	84 (42.21)	77 (46.11)		
>8 h	57 (15.57)	46 (23.12)	11 (6.59)		
Hormonal therapy^b^				12.885	<0.001***
>8 h	152 (41.53)	100 (50.25)	52 (31.14)		
<8 h	214 (58.47)	99 (49.75)	115 (68.86)		
Spinal segment fractured^b^				37.373	<0.001***
NO	307 (83.88)	145 (72.86)	162 (97.01)		
YES	59 (16.12)	54 (27.14)	5 (2.99)		
Treatment option^b^				7.774	0.005**
Conservative	84 (22.95)	34 (17.09)	50 (29.94)		
Surgery	282 (77.05)	165 (82.01)	117 (70.06)		
Rehabilitation^b^				1.572	0.210
NO	131 (35.79)	65 (32.66)	66 (39.52)		
YES	235 (64.21)	134 (67.34)	101 (60.48)		
Smoking^b^				0.011	0.917
NO	217 (59.29)	117 (58.79)	100 (59.88)		
YES	149 (40.71)	82 (41.21)	67 (40.12)		
Drinking^b^				0.157	0.692
NO	225 (61.48)	120 (60.30)	105 (62.87)		
YES	141 (38.52)	79 (39.70)	62 (37.13)		
Diabetes^b^				0.139	0.709
NO	315 (86.07)	173 (86.93)	142 (85.03)		
YES	51 (13.93)	26 (13.07)	25 (14.97)		
Hypertension^b^				0.848	0.357
NO	271 (74.04)	143 (71.86)	128 (76.65)		
YES	95 (25.96)	56 (28.14)	39 (23.35)		
Hyperlipidemia^b^				1.237	0.266
NO	291 (79.51)	163 (81.91)	128 (76.65)		
YES	75 (20.49)	36 (18.09)	39 (23.35)		

*Notes*: “*” indicates statistically significant difference (* *P* < 0.05, ** *P* < 0.01, *** *P* < 0.001)

Abbreviations: AIS, American Spinal Injury Association Impairment Scale; BMI, Body Mass Index; ISI, increased signal intensity; MSCC, maximum spinal cord compression; OPLL, ossification of the posterior longitudinal ligament

^a^ Median (25th, 75th)

^b^ Percentage (%).

### Feature Selection

**Fig. 6:**
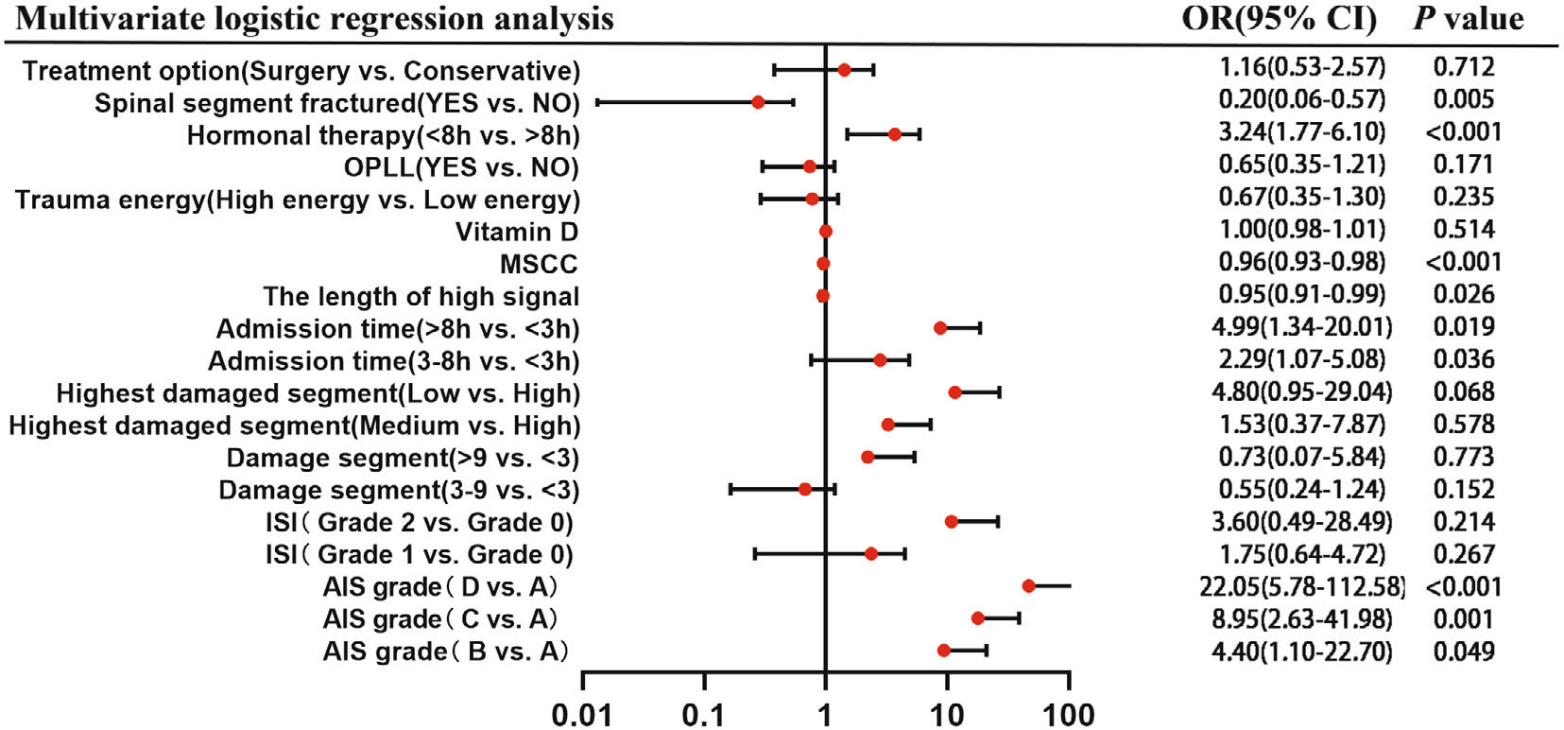
The forest plot shows the results of multivariate logistic regression analyses. In the multivariate logistic regression model, six independent influence factors for recovery were further screened out, including pre‐admission AIS grade (OR = 4.40, 95%CI 1.10–22.70, *P* = 0.049; OR = 8.95, 95% CI 2.63–41.98, *P* = 0.001; OR = 22.05, 95%CI 5.78–112.58, *P* < 0.001), admission time (OR = 2.29, 95%CI 1.07–5.08, *P* = 0.036; OR = 4.99, 95%CI 1.34–20.01, *P* = 0.019), The length of high signal (OR = 0.95, 95%CI 0.91–0.99, *P* = 0.026), MSCC (OR = 0.96, 95% CI 0.93–0.98, *P* < 0.001), Spinal segment fractured (OR = 0.20, 95%CI 0.06–0.57, *P* = 0.005), and hormonal therapy within 8 h after injury (OR = 3.24, 95%CI 1.77–6.10, *P* < 0.001). MSCC, maximum spinal cord compression

The univariate analysis was combined with a univariate logistic regression analysis to reveal factors influencing neurological recovery in CTSCI patients. Age (*P* = 0.075), sex (*P* = 0.851), BMI (*P* = 0.092), smoking (*P* = 0.833), drinking (*P* = 0.614), diabetes (*P* = 0.600), hypertension (*P* = 0.299), hyperlipidemia (*P* = 0.215), the number of sagittal T2‐WI MRI image (*P* = 0.071), and rehabilitation (*P* = 0.173) were not statistically significant in univariate logistic regression analysis. A total of 13 factors, the length of high signal in the spinal cord, MSCC, ISI, damage segment, highest damaged segment, OPLL, spinal segment fractured, Vitamin D, trauma energy, AIS grade, admission time, hormonal therapy within 8 h after injury, and treatment option, were included in the multivariate logistic regression model according to the test levels we set (Table 2 and Fig. 6).

**TABLE 2:** Logistic regression analysis to assess the factors influencing neurological satisfactory recovery in the training cohort

Factor	Univariable analysis				Multivariable analysis			
	β	OR	95% CI	*P* value	β	OR	95% CI	*P* value
Age	−0.02	0.98	0.97–1.00	0.075				NI
BMI	−0.05	0.95	0.89–1.01	0.092				NI
Sex								NI
Female	Ref	Ref	Ref	Ref				
Male	−0.05	0.95	0.59–1.55	0.851				
Rehabilitation								NI
NO	Ref	Ref	Ref	Ref				
YES	−0.30	0.74	0.48–1.14	0.173				
Smoking								NI
NO	Ref	Ref	Ref	Ref				
YES	−0.05	0.96	0.63–1.45	0.833				
Drinking								NI
NO	Ref	Ref	Ref	Ref				
YES	−0.11	0.90	0.59–1.34	0.614				
Diabetes								NI
NO	Ref	Ref	Ref	Ref				
YES	0.16	1.17	0.65–2.12	0.600				
Hypertension								NI
NO	Ref	Ref	Ref	Ref				
YES	−0.25	0.78	0.48–1.25	0.299				
Hyperlipidaemia								NI
NO	Ref	Ref	Ref	Ref				
YES	0.32	1.38	0.83–2.30	0.215				
AIS grade								
A	Ref	Ref	Ref	Ref				
B	2.13	8.40	2.50–38.55	<0.001	1.48	4.40	1.10–22.70	0.049
C	2.85	17.24	6.00–73.01	<0.001	2.19	8.95	2.63–41.98	0.001
D	4.21	67.68	22.92–291.56	<0.001	3.09	22.05	5.78–112.58	<0.001
ISI								
Grade 0	Ref	Ref	Ref	Ref				
Grade 1	−1.23	0.29	0.14–0.57	<0.001	0.56	1.75	0.64–4.72	0.267
Grade 2	−2.37	0.09	0.03–0.29	<0.001	1.28	3.60	0.49–28.49	0.214
Damage segment								
<3 segments	Ref	Ref	Ref	Ref				
3–9 segments	−1.75	0.17	0.10–0.29	<0.001	−0.60	0.55	0.24–1.24	0.152
>9 segments	−3.56	0.03	0.01–0.10	<0.001	−0.32	0.73	0.07–5.84	0.773
Highest damaged segment								
High	Ref	Ref	Ref	Ref				
Medium	1.93	6.89	2.66–23.56	<0.001	0.43	1.53	0.37–7.87	0.578
Low	3.07	21.50	7.27–80.56	<0.001	1.57	4.80	0.95–29.04	0.068
Admission time (h)								
<3	Ref	Ref	Ref	Ref				
3–8	−0.22	0.80	0.51–1.25	0.330	0.83	2.29	1.07–5.08	0.036
>8	−1.57	0.21	0.10–0.42	<0.001	1.61	4.99	1.34–20.01	0.019
The length of high signal	−0.07	0.93	0.91–0.95	<0.001	−0.05	0.95	0.91–0.99	0.026
MSCC	−0.06	0.94	0.92–0.95	<0.001	−0.04	0.96	0.93–0.98	<0.001
Vitamin D	0.01	1.01	1.00–1.02	0.040	0.00	1.00	0.98–1.01	0.514
Trauma energy								
Low energy	Ref	Ref	Ref	Ref				
High energy	−1.22	0.30	0.19–0.47	<0.001	−0.40	0.67	0.35–1.30	0.235
OPLL								
NO	Ref	Ref	Ref	Ref				
YES	−0.45	0.64	0.41–0.99	0.044	−0.44	0.65	0.35–1.21	0.171
Hormonal therapy (h)								
>8	Ref	Ref	Ref	Ref				
<8	0.80	2.23	1.46–3.45	<0.001	1.18	3.24	1.77–6.10	<0.001
Spinal segment fractured								
NO	Ref	Ref	Ref	Ref				
YES	−2.49	0.08	0.03–0.19	<0.001	−1.63	0.20	0.06–0.57	0.005
Treatment option								
Conservative	Ref	Ref	Ref	Ref				
Surgery	−0.73	0.48	0.29–0.79	0.004	0.15	1.16	0.53–2.57	0.712

*Note*: “*” indicates statistically significant difference. (* *p* < 0.05, ** *p* < 0.01, *** *p* < 0.001)

Abbreviations: BMI, Body Mass Index; MSCC, maximum spinal cord compression; AIS, American Spinal Injury Association Impairment Scale; ISI, increased signal intensity; NI, not included; OPLL, ossification of the posterior longitudinal ligament; β, regression coefficient; OR, odds ratio; 95% CI, 95% confidence interval.

Independent factors affecting neurological function recovery were further screened, including AIS grade (OR = 4.40, 95%CI 1.10–22.70, *P* = 0.049; OR = 8.95, 95% CI 2.63–41.98, *P* = 0.001; OR = 22.05, 95%CI 5.78–112.58, *P* < 0.001), admission time (OR = 2.29, 95%CI 1.07–5.08, *P* = 0.036; OR = 4.99, 95%CI 1.34–20.01, *P* = 0.019), The length of high signal (OR = 0.95, 95%CI 0.91–0.99, *P* = 0.026), MSCC (OR = 0.96, 95% CI 0.93–0.98, *P* < 0.001), Spinal segment fractured (OR = 0.20, 95%CI 0.06–0.57, *P* = 0.005), and hormonal therapy within 8 h after injury(OR = 3.24, 95%CI 1.77–6.10, *P* < 0.001). The remaining seven variables lacked statistical significance (Table 2 and Fig. 6).

### Establishment and Validation of the Nomogram

**Fig. 7:**
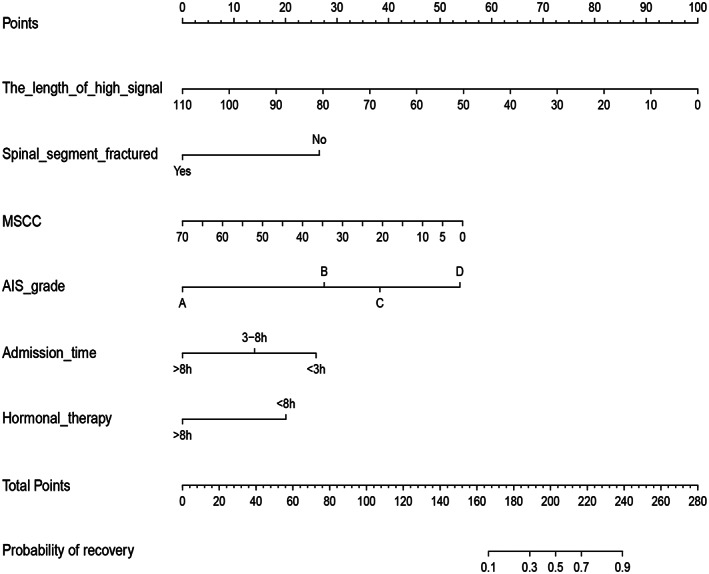
Predicting neurological function in CTSCI patients in a training cohort satisfactory recovery nomogram. Six independent predictors were involved in this model and a graph score was assigned to each predictor. The sum of these six scores produces a graph on the “Total Point” axis. The individual probability of satisfactory neurological recovery is summarized by drawing a vertical line from the “Total Point” axis to the “probability” axis. AIS, American Spinal Injury Association Impairment Scale. MSCC, maximum spinal cord compression

**Fig. 8:**
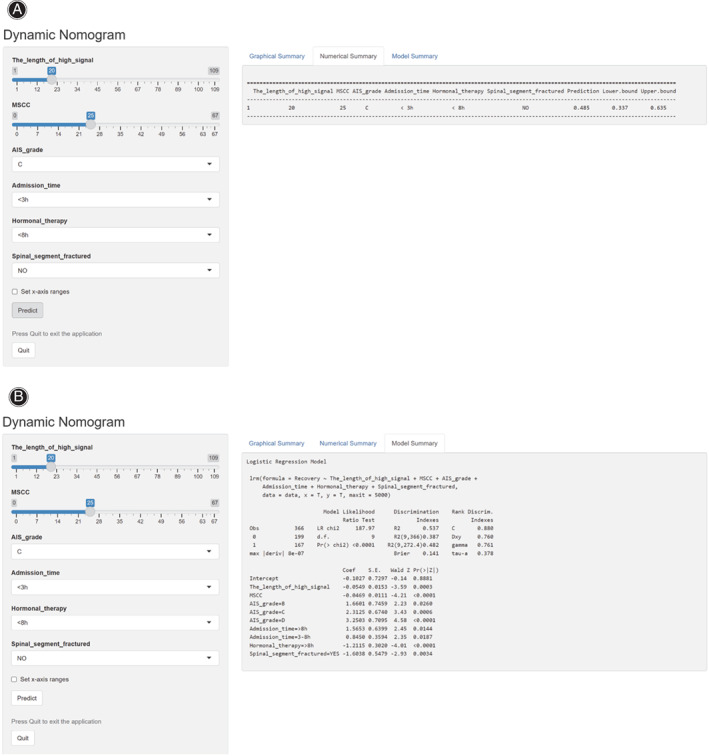
The online calculator converted from nomogram for generating probabilities of satisfactory recovery of neurological function. A Numerical summary of prediction. B Model details of prediction. AIS, American Spinal Injury Association Impairment Scale. MSCC, maximum spinal cord compression

A Nomogram prediction model was developed based on the six independent influences screened by the multivariate logistic regression model (Fig. 7). Each influencing factor in the nomogram is assigned a corresponding score. Each influencing factor was scored as appropriate for each individual patient, and the scores were then summed to obtain a total score. Based on the final total score, the probability of a satisfactory recovery of neurological function for this patient after 6 months can be obtained. Based on the results mentioned above, a dynamic Web‐based calculator was developed (the access link is https://dynomogramrsci.shinyapps.io/DynNomapp1/). For example, suppose there is a patient with CTSCI whose the length of high signal is 20 mm, MSCC is 25%, AIS grade is C, admission time <3 h, hormonal therapy within 8 h after injury, no spinal segment fractured; the probability of a satisfactory recovery of neurological function in this patient would be 48.5% (95%CI 33.7–63.5) based on our model (Fig. 8A,B). The C‐index for the prediction nomogram was 0.872 (95%CI, 0.837–0.907), and the interval bootstrapping validation C‐index was 0.862.

**Fig. 9:**
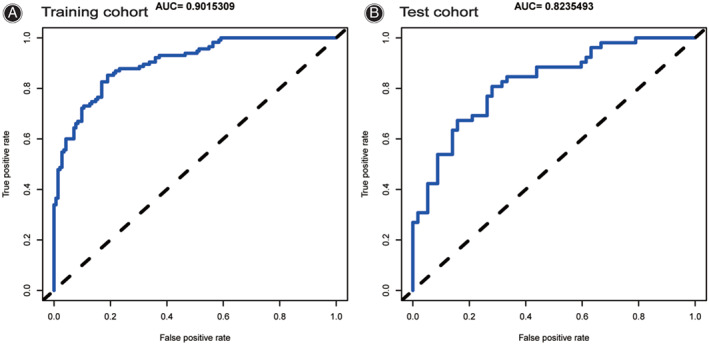
The AUC of the training cohort (AUC = 0.902) and test cohort (AUC = 0.824) indicate that the model had a high discrimination ability

**Fig. 10:**
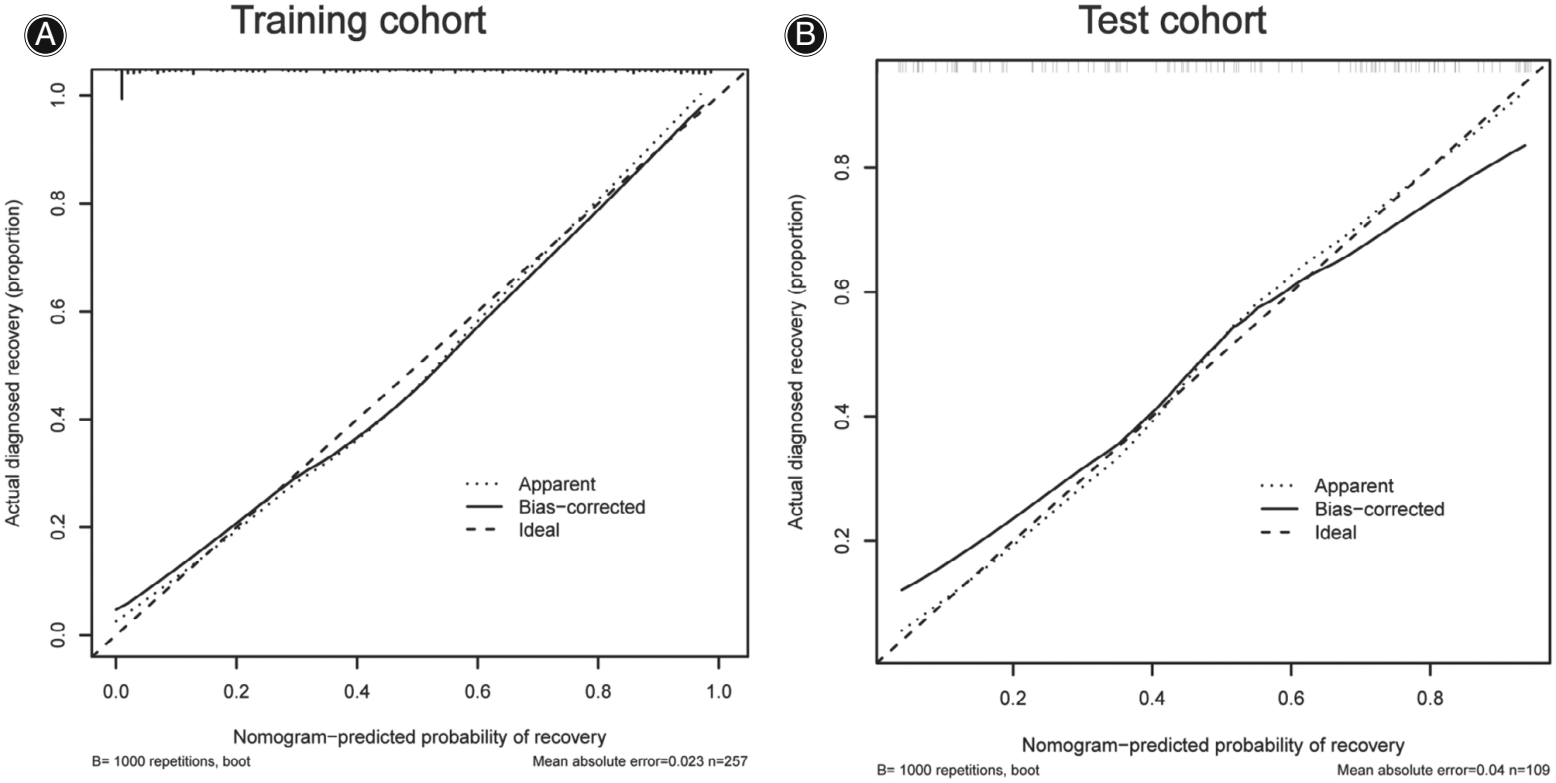
The calibration curve for assessing the consistency between the predicted and the actual probabilities of neurological function satisfactory recovery. There are favorable consistencies between the predicted and the actual probability assessments

We performed internal validation by including an additional 109 patients in the test cohort (Table 3). The ROC curves for the training and test cohorts were produced, and the AUC was computed to measure the prediction model's discrimination. The results demonstrated that the model had a strong capacity for discriminating. The AUCs of the training and test cohorts were respectively 0.902 and 0.824 (Fig. 9). Furthermore, calibration curves were created to illustrate the correlation between the anticipated value and the actual value. Both the training and test cohorts demonstrated a good connection between the anticipated likelihood of the nomogram and the actual circumstance (Fig. 10).

**TABLE 3:** Clinical characteristics of the internal test cohort

Factor	Total	Nonrecovery group	Recovery group
Number	109	57	52
The length of high signal (mm)^a^	14.00 [7.00, 27.80]	14.00 [7.00, 27.80]	14.41 [6.88, 27.90]
MSCC (%)^a^	30.85 [18.66, 39.14]	31.03 [19.10, 39.39]	30.85 [18.45, 39.27]
Hormonal therapy (h)^b^			
>8	46 (42.2)	27 (47.37)	19 (36.54)
<8	63 (57.8)	30 (52.63)	33 (63.46)
Spinal segment fractured^b^			
NO	93 (85.3)	44 (77.19)	49 (94.23)
YES	16 (14.7)	13 (22.81)	3 (5.77)
AIS grade^b^			
A	16 (14.7)	14 (24.56)	2 (3.85)
B	14 (12.8)	10 (17.54)	4 (7.69)
C	43 (39.4)	24 (42.11)	19 (36.54)
D	36 (33.0)	9 (15.79)	27 (51.92)
Admission time (h)^b^			
<3	41 (37.6)	19 (33.33)	22 (42.31)
3–8	57 (52.3)	31 (54.39)	26 (50.00)
>8	11 (10.1)	7 (12.28)	4 (7.69)

Abbreviations: AIS, American Spinal Injury Association Impairment Scale; MSCC, maximum spinal cord compression

^a^ Median (25th, 75th)

^b^ Percentage (%).

## Discussion

It is critical to comprehend the incidence of CTSCI and the factors influencing its prognosis. To this end, we analyzed clinical and imaging data from 475 CTSCI patients at our institution and found that six influencing factors, the length of high signal, MSCC, admission time, hormonal therapy, spinal segment fractured, and AIS grade, play a decisive role in the neurological recovery of CTSCI patients.

### Identification of Prognostic Factors

Previous studies have reported that recovery of neurological function in patients with CTSCI is influenced by a variety of factors, including ASIA grade, MSCC, the length of high signal in the spinal cord, age, admission time, treatment option, damage segment, hormonal therapy within 8 h after injury, and spinal segment fractured.^6^, ^18^, ^19^ However, the relative importance of these influences remains unclear. Therefore, this study constructs a user‐friendly predictive model based on the clinical characteristics of CTSCI patients and the factors affecting their prognosis to assess the prognosis and take appropriate interventions.

Based on previously published data, 23 potential impact variables were initially selected for this study. Logistic regression analysis found that six variables collected at our institution (admission time, MSCC, the length of high signal in spinal cord, hormonal therapy, AIS grade at admission, and spinal segment fractured) were significantly associated with satisfactory recovery of neurological function in patients with CTSCI. Salazar *et al*.^20^ found that the longer the time to injury before hospital admission, the worse the prognosis.

### Admission Time Affects the Prognosis of CTSCI

This may be due to the large number of nerve cell deaths that can occur within hours of CTSCI, as well as the longer the duration of injury before admission, the more delayed the standard treatment such as hormone therapy, dehydration therapy, and neck immobilization. This is in line with our study. Therefore, clinicians should treat patients with CTSCI immediately when they encounter them, as this is crucial to their prognosis.

### MSCC and The Length of High Signal in the Spinal Cord Affect the Prognosis of CTSCI

Miyanji *et al*.^5^ discovered that MSCC and the length of high signal in the spinal cord are significant predictors of neurological recovery in 100 patients with CTSCI. In addition, Heredia Gutiérrez *et al*.^21^ reported that the greater spinal cord compression, the more pronounced the decrease in spinal conduction function. Recovery is almost impossible if the spinal cord is compressed by more than 50%. In other studies, the prognosis is better the shorter the length of edema and bleeding in the cervical spinal cord on MRI following spinal cord injury. Boldin *et al*.^22^ revealed that the shorter the length of edema and hemorrhage in the cervical spinal cord on MRI following spinal cord damage, the better the prognosis. Our study also found that the MSCC and the length of the high signal in the spinal cord were factors affecting the prognosis of the patients and that the larger the MSCC and the longer the length of the high signal in the spinal cord, the worse the prognosis of the patients. Evaluation of individuals with CTSCI requires an MRI to image the damaged spinal cord and prognosis.^23^ Therefore, Miyanji *et al*.^5^ suggest MRI should be performed on all patients with cervical spinal cord injury whenever possible. With the increasing understanding of the pathophysiological changes following spinal cord injury, a number of neuroprotective treatments have been developed to reduce the secondary damage to the spinal cord.^8^

### Hormones Affect the Prognosis of CTSCI

Hormones may be an effective treatment modality for improving neurological function after spinal cord injury as they can influence the onset of a wide range of secondary injuries. It has been shown that high‐dose methylprednisolone shock therapy within 8 h of injury is moderately effective.^24^ If patients are diagnosed and treated within 3 h postoperatively, their outcome is better than if they are treated later (within 8 h), and patients who do not receive hormonal therapy within 8 h post‐injury have a poorer prognosis. This is also in agreement with our study. Therefore, frontline clinicians should give high‐dose hormone shocks to patients with CTSCI in the first instance, as this is critical to the patient's prognosis.

### AIS Grade Affect the Prognosis of CTSCI

Nakajima *et al*.^25^ analyzed 280 patients with cervical spinal cord injuries. 11.8%, 22.6%, and 62.3% of patients with AIS A, B, and C regained ambulation, and the severity of AIS at admission was the strongest predictor of this functional prognosis. Thompson *et al*.^6^ found that admission AIS grade affected patient prognosis. A systematic study revealed conversion rates to AIS D of 3%, 31%, and 67% for AIS A, B, and C cases, respectively, indicating that patients in the AIS A group have a low likelihood of walking recovery.^26^ Similarly, the admission AIS grade was an essential factor in our study's prognosis of patients with CTSCI.

### Spinal Segment Fracture Affects the Prognosis of CTSCI

A spinal segment fracture combined with a cervical spinal cord injury is a serious trauma that can result in death or disability.^27^ Currently, the best treatment for spinal segment fracture combined with cervical spinal cord injury is a surgical repositioning of the fractured spine with spinal cord decompression, which restores normal alignment and stability of the damaged segment and relieves spinal cord compression‐created conditions for neurological recovery.^28^, ^29^ Although the surgical technique for spinal segment fractures combined with cervical spinal cord injury is well established, the patient's postoperative recovery has not been satisfactory.^30^ In our study, only 5 (8.5%) of the 59 patients with spinal segment fractured combined with cervical spinal cord injury had satisfactory recovery of neurological function.

### Construction and Advantages of this Novel Clinical Model

Therefore, it is clinically appropriate to develop a simple and effective scoring model to predict the recovery of neurological function in patients with CTSCI after 6 months. Based on a multivariate logistic model, a nomogram can integrate all factors related to prognosis and comprehensively evaluate each factor's cumulative effect on patients, which is gradually applied to predict the likelihood of individual neurological recovery. Unlike the traditional nomogram, the dynamic nomogram in this study is easy to calculate with a simple and straightforward interface, which can quickly calculate the prognosis of different individuals. Furthermore, the high C‐indexes showed ideal validation of the prediction and demonstrated good model fit by ROC curves.

### Strengths and Limitations

This study has several advantages. This is the first nomogram to predict the probability of satisfactory neurological recovery in CTSCI patients 6 months after injury. Our nomogram predictors are routinely measured after each patient has been admitted to the hospital to refine their cervical MRI. Considering the data we have obtained in the acute phase of all CTSCI patients, clinicians can use the nomogram to identify high‐risk patients with poor prognoses early and provide individualized and precise treatment strategies. For the web‐based calculator, we have created a dynamic nomogram (the access link is https://dynomogramrsci.shinyapps.io/DynNomapp/), which is easier to use than the traditional nomogram by simply entering the data to get the predictions. At the same time, we recognize some limitations. All the patients in our study were from southeastern China, and the sample size was not large enough to represent the entire population of China and Asia. Therefore, a larger sample size and random selection of patients from China and even Asia will be essential to produce more convincing results in future studies. In this study, patients with CTSCI were followed up for only 6 months, which may be too short a follow‐up period. We had only performed internal validation and had not completed external validation, which would negatively impact the model's applicability. Our inclusion of influencing factors did not include all potential factors associated with the prognosis of patients with CTSCI.

### Conclusion

Overall, the nomogram developed by our team to predict the probability of satisfactory neurological recovery in CTSCI patients is of excellent practical value. In clinical practice, the predictive model can effectively help clinicians stratify patients and provide them with personalized treatment plans, which is conducive to improving patient satisfaction with treatment and enhancing the relationship between patients and clinicians.

## Author Contributions

Xin Yan Conceptualization, Methodology, Data curation, Writing‐original draft. Yaozhi He and Mengxian Jia: Data curation, Writing‐original draft. Jiali Yang and Peng Zhang: Investigation, Formal analysis. Kelun Huang, Jiaxin Lai, and Minghang Chen: Investigation, Formal analysis, Writing‐review & editing. Shikang Fan, Sheng Li and Ziwei Fan: Validation. Honglin Teng: Supervision, Project administration.
